# Progress of the Medical Sciences

**Published:** 1891-12

**Authors:** 


### Progress of the Medical Sciences.

### MEDICINE.

An excellent review of Intermittent or "Functional" Albuminuria has been contributed by Dr. J. W. Brannan,[1] who gives an account of the autopsy upon a typical case. Many cases of intermittent albuminuria have several factors in their causation, and classification must therefore be more or less arbitrary; but the following scheme of subdivision is propounded:—Class I., Dyscrasic; Class II., Mechanical; and Class III., Neurotic.

The first class includes the early stages of Semmola's dyscrasic condition, especially hetero-albuminæmia; albuminuria due to derangements of the gastro-intestinal and hepatic viscera, that due to gout, that associated with glycosuria, and probably that following a cold bath. The second class comprises the albuminuria of adolescents, or "cyclical" (Pavy). This is found at all periods of life, and apparently depends principally upon position. On rising in the morning the weight of blood resting on the renal veins is suddenly increased, and albumin appears in the urine in one or two hours, and gradually disappears during the day. The albuminuria does not appear so long as the patient remains in bed. Herringham found that there was no relation between this albuminuria and diet on the one hand, or the excretion of urea and uric acid on the other. Heubner, who observed this condition in three sisters, found that even forced movements do not induce it, provided that the patient remains in the recumbent posture. The third class is composed of those cases of albuminuria following anxiety or intellectual effort, such as passing examinations. Sir A. Clark has seen it repeatedly follow political speaking in persons at other times free from it. Perhaps also those cases of albuminuria due to excessive physical exercise should be included here, as both this and the preceding form are due to weakened state of renal vaso-motor centres. Dr. Goodhart suggests that in such cases there may be a visceral flux, comparable to that which takes place about the head and face of a neurotic

[1] *New York Med. Journ.*, July 4, 1891.

woman; and thinks that they give support to Dr. Clifford Allbutt's view, that granular kidney dates its origin from a period of anxiety or worry. Dr. Brannan thinks that all varieties of functional albuminuria, unless arrested, will result ultimately in change of renal structure. Albuminuria is always pathological, and the nutritive changes in the glomerular epithelium may be slight or only temporary, but not the less abnormal. Lecorché and Talamon believe it to be caused by glomerulitis, limited to a few glomeruli at a time. In the case of "functional" albuminuria, in which an autopsy was obtained after death by suicide, eight specimens of urine had been examined during a preceding period of six months: of these, seven were absolutely normal in every way; in the remaining specimen, a slight amount of albumin was discovered after filtering the urine, but no casts of any kind. The epithelium of the convoluted tubules showed fatty degeneration, the glomerular capsules were thickened by fibrous tissue in concentric layers, and here and there were small areas of round-cell formation. A man, therefore, in *apparent* perfect health, whose infrequent albuminuria would fairly have been considered functional, showed commencing nephritis as well as marked commencing degeneration of the vascular system. Such a case carries with it a significant warning.

The relation of albuminuria to life insurance is likely to become a highly serious question. Several insurance companies are proposing to abolish the medical examination of candidates for insurance, apparently as a matter of economy or parsimony. Now, it is evident that a person, the subject of an unknown albuminuria, may, with unimpeachable *bona fides*, declare himself to be in sound health to the best of his belief, although in reality far advanced in parenchymatous or interstitial nephritis. An extreme instance came under my own notice lately. At the discussion at the annual meeting of the British Medical Association in 1889, of the fifteen distinguished physicians who took part in the debate, five favoured the acceptance of persons with albuminuria, who were otherwise healthy, at advanced rates, or for short terms; five others advised postponement until the disappearance of the albumin, and only two advocated rejection outright, among the first being Pavy, Johnson, and Saundby. Harley attaches more importance to the specific gravity than to the presence of albumin or casts; Tyson thinks life assurance companies, in justice, ought to recognise a condition of purely functional albuminuria. Grainger Stewart says that the existence of albuminuria is not of itself a sufficient ground for rejection; that tests have demonstrated albuminuria to be present in one-third of the population; and that Bright's disease does not account "but for a fraction" of the cases of albuminuria. Among persons applying for life insurance, Dr. Brannan found 14 per cent. had more or less albumin in their urine, although in not more than 5 per cent. did he find albumin without other symptoms of Bright's disease. Rabagliati considers that in a case with only a trace of albumin, no casts or other definite lesion, the life may be taken as having an expectation of attaining its fifty-seventh year. Pollock proposes acceptance for five years, with re-examination upon the expiration of that period. The latest authoritative exposition on the subject is by Dr. W. B. Davis of Cincinnati,[1] who gives a code of stringent rules applicable to such candidates, one of these rules being that there must be a period in the twenty-four hours when the urine is free from albumin. He concludes his remarks by saying that when the conditions of these rules are met, the candidate may be accepted for a short endowment policy; for until the clinical significance of albuminuria in persons apparently healthy has been finally determined by observations upon one full generation, we cannot, and probably ought not, to expect the assurance companies to do any better for them.

Those who have been carefully making repeated examinations of the acidity of the contents of patients' stomachs during digestion, must have been disappointed by the capricious variability of the results—a variability so great as considerably to impair the validity of the test as a means of diagnosis. It is generally agreed that the colour reactions with phloroglucin-vanillin and benzo-purpurin are trustworthily indicative of the presence of free hydrochloric acid in the gastric secretion. Constantly one finds no reaction for free acid when there is nothing obviously abnormal in the stomach and its functions. Recently published researches may perhaps explain this apparent contradiction. Hayem and Winter[2] lay great stress on the conclusions of Richet, that the principal part of the hydrochloric acid is secreted and contained in the stomach as organic compounds of chlorine. All previous methods they condemn, as not taking into consideration these organic compounds of chlorine; and the clinical results of Ewald and Boas they think illusory for

[1] *Medical Record*, June 13, 1891. [2] *Du Chimisme Stomacal.*

the same reason. Salkowski considers that he has proved that the digestive power of the gastric secretion was the same whether the hydrochloric acid was combined with the amido-acids, or whether the fluid was an equally strong hydrochloric acid pepsin solution. Rosenheim,[1] though not agreeing with Salkowski that solutions of hydrochlorate of leucin are equally as potent as free hydrochloric acid solutions containing the same amount of pepsin, admits the diminution of digestive power to be but slight in the case of the amido-acids, though very considerable in the case of albumoses and peptones. Rosenheim also found that not infrequently in natural digestion the function went on in the absence of free hydrochloric acid, though the more abundant the acid the more rapid the process. Saundby[2] records a case of fatal perforating gastric ulcer, whose stomach-contents during life gave no evidence of free hydrochloric acid. Most physicians have seen similar cases. Obviously what we now want is an easily-applied test, which will show hydrochloric acid in organic combination.

Bacteriologists are still divided into two sections: those who hold that microbes gaining entrance into a living body are destroyed by the leucocytes, and those who believe that the microbes are destroyed by the blood-serum and body-fluids in general. The contention almost daily receives additional arguments and experimental evidence upon both sides, and undoubtedly the preponderance of opinion is toward the cell-destroying action of the phagocytes, as Metschnikoff denominates the leucocytes in this particular capacity. Still, it is clear that the theory of phagocytosis is by no means totally adequate to explain immunity, congenital or acquired. Burdon Sanderson, in his Croonian lectures, is careful to limit the meaning and importance of "the unquestionable fact of phagocytosis," and assigns to it a very secondary place in the mechanism of immunity. Metschnikoff himself, in a *critique*[3] upon tuberculin, admits that the leucocytes "incept," but do not destroy the tubercular bacilli; all that they can do is to hinder their development, and so it is comprehensible that the phagocytes, unable to destroy the bacilli, become the means of propagating the disease in other regions, and of transforming a localised into a general malady; passing along the lymphatics, and so into the blood, they become located in a position distant from

[1] *Centralblatt für klin. Med.*, Sept., 1891. [2] *Birmingham Med. Review*, Oct., 1891.

[3] *Annales de l'Institut Pasteur*, No. 3, 1891.

inflammatory action, and serve as foci of new tubercular growths. "Inception" therefore is completely different from destructive phagocytosis. Finger [1] has shown that the bacilli of glanders, injected into non-susceptible animals, like white mice, rapidly die and disappear without any intervention on the part of the leucocytes, destroyed by the microbicidal properties of the fluids of the tissues. Last year Behring and Kitasato showed that cell-free blood-serum, taken from animals which had been rendered immune to tetanus and diphtheria, was capable of curing other animals suffering from those diseases. Recently G. and F. Klemperer have shown[2] that the same holds good for pneumonia; serum taken from animals enjoying immunity was found capable of curing pneumonic septicæmia. Various other such examples could be adduced. These bactericidal and immunity-conferring substances are termed defensive proteids by Hankin, and alexins by Buchner. Of what nature they are is not exactly known, beyond that they are either globulins, or inseparably connected with the globulin of the blood. Adami's[3] opinion, constituting a kind of reconciliation between the two opposing theories, is that they are derived from, or elaborated within, facultative phagocytes, and are found in the serum owing to the breaking down of those corpuscles. It is almost certain that there must be a considerable number of these defensive proteids, though in this connection Burdon Sanderson speaks of *a* substance contained in the liquor sanguinis, inasmuch as the blood-serum which confers immunity against one infective disease does not do so against any other. Some trials have been made in the treatment of pneumonia in the human subject upon this method of therapeutics, but at present we wait with eagerness the result of further experiment.

Tuberculosis being the commonest serious disease known to be due to an infective organism, experiment and therapeusis have of late been mainly directed by bacteriologists to the investigation of that malady. Merely to relate the nature of the different "cures" which have been introduced within the last few months would alone more than occupy the space available for this Report. With perhaps the exception of Lannelongue's treatment of tubercular joints by the injection of solution of chloride of zinc into the immediate periphery of the articulation, thereby inducing a sclerogenic process,

[1] *Wien. med. Wochen.*, No. 19, 1891. [2] *Berl. klin. Wochen.*, Aug. 24, 31, 1891.
[3] *Medical Chronicle*, May, 1891.

probably none of the methods brought forward during the present year possess advantage over processes known before. Koch published a few weeks ago another communication on tuberculin, of which the opening remarks are regrettably peevish in tone, and the details given in the body of the manifesto greatly disappointing. His so-called "pure" tuberculin, when used in the Moabit Hospital, was found not to differ appreciably in its action from the crude substance, the diagnostic and therapeutic effects of both being the same. Still more recently Klebs has published [1] his own researches, and certainly has been more successful than Koch in separating the various active constituents present in crude tuberculin. The remedial substance he finds to be an albumose, never exciting fever; and under its use the bacilli in the sputum become granular, less and less capable of taking staining reagents, and finally disappear. It is well to observe that Koch himself, Klebs, and Ehrlich now only make use of very small doses of tuberculin, carefully avoiding the production of the injurious "reaction," so sought for at first. Klebs, indeed, is far from having approached Hunter and Watson Cheyne in investigating the nature and actions of the component principles of tuberculin. There is no need to call attention to the researches of our own countrymen as being of intense interest, and probably of intense importance; but, although their results are corroborated, their work is absolutely ignored by their continental *collaborateurs*. Hueppe has quite lately vigorously criticised [2] Koch, his methods and his communications, justly upbraiding him with ignoring the equal knowledge both of his predecessors and contemporaries. Partly deplorable and partly unsatisfactory as all this may be, it will in time help to guide us in our track towards immunity—the El Dorado for which as yet, in the case of tubercle, we are only able to hope.

If it is possible to show more completely how impotent the Lunacy Act of 1890 is to protect medical men against unjust actions for damages for having signed certificates under the Act, two cases, which have lately been tried, give plenary proof thereof. In the one case a doctor gives a statement about a man, who is his regular patient, and who had already been in an asylum for suicidal melancholia, to the effect that he is of unsound mind. This statement was given for the purpose of being taken to the relieving officer, by whom it was in due course employed to effect the man's

[1] *Deutsch. med. Wochen.*, Nov. 5, 1891. [2] *Berl. klin. Wochen.*

removal to a workhouse infirmary. A year after the man's discharge he brings an action against the doctor, and obtains a verdict of £25 damages. The doctor had undoubtedly made the error of not seeing his patient immediately before giving the statement; but the judge, while ruling that the statement was not a libel, imported collateral issues into his summing-up, and upon them it must be presumed the verdict was given. The other case is of a much more iniquitous nature, as these supposedly protective clauses were deliberately violated. Dr. Alfred Carpenter and Dr. Dukes, of Croydon, have recently emerged from the trial of an action, which had been hanging over their heads for two years, with damages laid at £2,000 against each of them. First of all, directly contrary to the provisions of sec. 331, more than a year had elapsed after the discharge from confinement of the plaintiff before the action was commenced; and, secondly, under sec. 330, sub-sec. ii., application was made to the High Court to stay proceedings, but without success. It is of special interest in this case to note that the Counsel for the defence argued his case entirely on the basis of the reasonable care and good faith of the defendants, and did not attempt to prove the insanity of the plaintiff, as hitherto it has always been thought necessary to do. This is a distinct gain to the medical profession, so far as it goes. In the end a verdict with costs was entered for the defendants; but, as usually happens, the plaintiff is impecunious, and our respected colleagues, besides the worry and anxiety endured, are subjected to a heavy pecuniary loss for legal expenses. Although the case has been commented upon by the leading medical journals, it is a matter for regret to see that nothing has been done towards raising a fund by which to render some compensation to Drs. Carpenter and Dukes. Under circumstances so peculiarly harassing, it is a sincere consolation to receive sympathy and help from one's professional brethren. *Experto crede!*

Since the above was set in type it is satisfactory to note that the plaintiff's appeal for a new trial has been dismissed, and that some compensation to the defendants is likely to result from a letter by Dr. Carpenter in the *Lancet* of Dec. 12.

J. E. Shaw.

Now that another epidemic of influenza is upon us, a consideration of the official report of the Epidemic of 1889-90, by Dr. Franklin Parsons, is of great interest.

Although bacteriologists have not been able to associate the disease uniformly with any specific germ, the diplococcus pneumoniæ found to be frequently present, and the streptococcus pyogenes even more constantly, both being probably results rather than the cause of the disease, the facts given by Dr. Parsons show:

1. That the progress of the epidemic was contrary to the prevailing winds, and that it was independent of season or any particular kind of weather;

2. That it has not been shown to have travelled faster than human beings could travel;

3. That it has not occurred among persons placed under circumstances precluding its communication by human agency;

4. That as a general rule in each country it has appeared first in the capital, or the ports of entry, or the frontier towns in communication with countries previously invaded, and that the towns, as a rule have been affected earlier than country places;

5. That neighbouring communities have in certain instances been affected only at considerably different dates;

6. That many instances are recorded of the disease having been introduced into a district, and spread to persons in contact with the patient, and sometimes afterwards to others;

7. That persons brought much into contact with others, *e.g.*, people going daily to business in towns, have generally been the first to suffer; their households, and people locally employed, being affected later;

8. That in public services and establishments persons employed together in large numbers in enclosed spaces have suffered in larger proportions than those employed few together, or in the open air;

9. That in institutions in which the inmates are brought much into association the epidemic has more quickly attained its height, has prevailed more extensively, and been sooner over, than in those in which the inmates are more secluded from one another;

Hence Dr. Parsons is of opinion "that the epidemic has been propagated mainly, perhaps entirely, by human intercourse."

Dr. Buchanan's judgment on the question is also very decided, as he writes: "Probably no evidence has ever been put on record in such abundance as that accumulated in Dr. Parsons's report, to show that in its epidemic form influenza is an eminently infectious complaint, communicable in the ordinary personal relations of individuals one with another. It appears to me that there can henceforth be no doubt about the fact."

The following from Dr. Bezly Thorne will be of interest: "I believe that influenza is an infectious and febrile affection of the cerebro-spinal nerve centres: that the *materies morbi* enters the system through the conjunctiva, in which structure it probably incubates its infecting brood, and thence passes through the tissues of the eye and optic nerve into the encephalon and spinal cord: that the overflow of the tears carries the infection through the nasal duct into the nares, which may or may not pass into a state of acute catarrh according to the state of health in which they happen to be at the time: that from the nares infecting material passes from the post-nasal region into both the œsophagus and larynx, and that affections of the respiratory or gastro-intestinal systems may ensue, or not, according to whether they present the conditions which are favourable to the development of acute catarrhal affections."

R. SHINGLETON SMITH.

### SURGERY.

Dr. R. W. Taylor [1] advances some excellent reasons for disbelief in the utility of excision of the chancre as an abortive treatment of syphilis. He records three cases of his own, where the initial lesion was freely excised on the first day of its appearance, and in each case constitutional symptoms of syphilis followed after the usual interval. In one of the cases, a careful microscopical examination of the parts adjacent to the chancre was made by Dr. Van Gieson, who found a remarkable change in the blood-vessels for a considerable distance around the lesion. This change in the blood-vessels consisted in the distension of the perivascular spaces with small round cells, the masses of cells forming a sheath for the blood-vessels like a coat-sleeve around the arm. Besides this condition of the perivascular spaces, there was a change in the endothelial cells lining the arteries and veins, the endothelial cells being swollen and proliferating. The microscope thus showed how deep-rooted syphilis is at the very beginning of the sore, by having propagated itself along the perivascular lymph spaces, and how futile it is to attempt to stay syphilis by excising the primary sore.

Dr. Taylor's studies go to show that in the very first days of syphilitic infection, as shown by the chancre after the first period of incubation, the poison is deeply rooted beneath the

[1] *Medical Record*, July 4, 1891.

initial lesion, and that it extends far beyond it; that it is in a most active state, and running along the course of the vessels, it soon infects all the parts beyond, even to the roots of the penis. He describes the consensus of opinion among advanced syphilographers of to-day as being that the operation is utterly futile; and he especially quotes Ricord, who in his later years said that he considered the destruction of the infecting chancre as absolutely useless (in a prophylactic sense), no matter how early it is done; that it is certain that even before its appearance syphilis exists, and that even if the entire penis should be amputated before the chancre showed itself, syphilis would follow nevertheless.

This view is strongly combated by Dr. S. Pollitzer of New York.[1] He maintains that the observations of Auspitz, Unna, Pick, and many others, go to show that it is sometimes possible to remove the primary syphiloma entirely; while in other cases excision of the syphilitic growth can be at least partial.

The bacteriological researches of Bouchard and Chauveau have demonstrated that the intensity of infection is (within limits) directly dependent on the number of pathogenic organisms. If, therefore, we remove the reservoir of infection by excising the chancre promptly, we are helping the organism in its struggle against the invader, and there is at least a theoretical possibility that the intensity of the infection will be mitigated. That this is actually the case there is a considerable amount of evidence. At the Berlin Congress, Ehlers of Copenhagen reported thirty-seven excisions in which the patients had remained under observation for several years. In only ten per cent. of these did severe forms of syphilis develop, whereas the proportion of cases of severe syphilis in general is about thirty-five per cent.

Of fifteen cases upon which Jullien operated, three showed absolutely no signs of syphilis later, and in a fourth the disease was so attenuated—if not wholly eradicated—that the patient acquired later a second syphilitic chancre. Leloir, in a recent paper, reports several cases in which absence of all symptoms followed after excision of the chancre.

From these and similar cases, Dr. Pollitzer thinks we are warranted in saying that there is some probability that a syphilis may be aborted by the early excision of its initial lesion, and a considerable probability that its course will be rendered more benign.

M. Fournier, in the *Gazette des Hôpitaux*, 24th September, 1891, gives a very good summing-up of our present knowledge on the subject. He points out that the old methods of trying

[1] *Medical Record*, Oct. 24, 1891.

to arrest the disease by cutting the lymphatics or by destroying the chancre by caustics have been completely given up, and the only plan ever now adopted is excision of the chancre.

This should be done by raising it well up from its bed by means of a tenaculum, and then cutting widely around it with a bistoury, removing as much of the surrounding tissue as we should do in excising a malignant growth. Removal of the chancre after induration has set in, or after the glands have become affected, is perfectly useless, and even if done at a very early stage the prospect of a favourable result is only slight. He quotes a number of recently-published statistics, which would indicate that, under favourable conditions, one success in five cases may be looked for; but he points out that statistics are of very little value. For in order to prove anything, each published case ought to include four conditions: 1st. There should be an examination of the source of infection, so as to make certain that the disease could be caught. 2nd. The duration of the incubation of the chancre should be recorded. 3rd. A full description of the lesion should be given, and not a mere statement that it was a hard chancre. And, 4th, a prolonged observation of the case should follow, without any treatment by mercury or iodine. He sums up by saying that we ought to give patients the benefit of the chance afforded by the operation, even although the chance be a small one.

M. Diday, in the *Gazette des Hôpitaux*, 13th October, 1891, gives his views of the abortive treatment of gonorrhœa in the male. He maintains, 1st, that in the early stage all gonorrhœa is abortible; and, 2nd, that the abortive treatment is not dangerous, locally or constitutionally. It is necessary to act as soon as inflammation shows itself at the meatus, but it is rather late when the meatus is œdematous, and it is quite too late when a purulent discharge has become established. One of two methods may be adopted: a strong solution may be introduced into the urethra and allowed to remain a short period, or a weaker solution may be used for a longer period. He generally uses a 5 per cent. solution of nitrate of silver, and varies the period of duration by the pain set up, but it should remain in the urethra 40 seconds at the least, a minute and a half at the most. He introduces the solution with a syringe, and then applies a finger to the orifice of the meatus, not squeezing it, for this would prevent contact with the navicular fossa. He then moves the fluid up and down the urethra by pressure upon the outside, thus ensuring the requisite amount of penetration. He finishes by what he calls the toilet of the meatus: the penis being held vertically, a few drops of the solution are instilled between the lips of the meatus. After an interval of two hours, a copious discharge takes place. It is not until four days afterwards that we can judge of the success of the treatment. In some cases the cure is then complete, in others a purulent discharge persists; but if this be due merely to the caustic, it will pass off in two or three days further.

Considering the grave complications which sometimes follow this malady, he thinks that the abortive treatment is well worth a trial.

Hydrocele is a very common disease in India, and the *Indian Medical Gazette* for July, 1891, gives a useful summary of the best modes of treatment. In ordinary cases, nothing can be better than the injection of tincture of iodine. It is very safe, and provided that the tincture be strong and that careful manipulation ensures its application to the whole inner surface of the distended tunica, it seldom fails.

The only objections to it are, the pain it causes, the pyrexia it sometimes sets up, and the iodism which in some cases results from its absorption. The primary pain can be prevented by a preliminary injection of morphia, or still more completely by throwing into the sac, after removing the fluid, a few drachms of a 10 per cent. solution of cocaine, allowing it to remain there for ten minutes or so, and letting it escape before introducing the iodine.

Treatment by injection of strong carbolic acid is recommended by Dr. S. E. Milliken [1] of New York. He has used it in 54 cases; and of these, 9 were never seen after the injection; 5 were seen once only, during the first week; and 4 were were still under observation. The remaining 36 were all cured; 27 had one injection, 4 had two injections, and 5 had three injections. In no case did sloughing occur, and not one of the 36 patients lost more than twenty-four hours from business.

He evacuates the hydrocele fluid through a small trocar, and then screws on to the cannula a hypodermic syringe, containing 5 to 25 minims of liquefied crystals of carbolic acid, taking great care not to allow a drop of the acid to come in contact with the skin of the scrotum. After removal of the cannula, slight kneading of the sac is done, to ensure thorough coating of its walls with the irritant.

The advantages claimed for this method of treatment are:

[1] *Annals of Surgery*, Oct., 1891.

(1) it is safe; (2) it is practically painless; (3) the patient is allowed to attend to business without more than one day's delay; (4) the use of an anæsthetic is avoided.

Treatment by electricity is advocated by Assistant-Surgeon Nundo Lall Ghose[1] of Dacca. He first draws off the hydrocele fluid by an ordinary trocar and cannula, and when the sac is empty an insulated platinum probe, attached to the negative pole of a battery, is passed through the cannula and freely applied to nearly the whole of the inner surface of the sac, with a current of about four milliampères, for about three minutes, the positive pole being placed over the groin. Three cases out of four treated by this method were successful.

Volkmann recommends incision and drainage; and Von Bergmann advises excision of the tunica vaginalis. This last is, no doubt, very effectual, and where asepsis is maintained it is quite safe and comparatively painless. But if the antiseptic measures fail, and they are very prone to do so in this locality, painful and serious consequences may result.

At the German Surgical Congress, in 1890, it was resolved that members of the Society should make a collective investigation of the cases in which they administered anæsthetics during the period between July 1st and December 31st, 1890. Sixty-six members of the Society sent in reports of their cases, and Professor Gurlt summarises them in a paper published in the *Archiv für klin. Chirurgie*, and quoted by the *Medical Chronicle*, September, 1891.

The cases are 24,625 in number, and the anæsthetics used were as follows:

| | | |
|---|---|---|
| 22,625 | chloroform . . . . | 6 deaths |
| 470 | ether . . . . . . | No death |
| 1,055 | ether and chloroform . . | No death |
| 417 | ether, chloroform, and alcohol | No death |
| 27 | ethyl bromide . . . . | No death |
| 24,625 | | 6 deaths. |

It will be seen that all the deaths occurred from chloroform, in the proportion of 1 to 3,776 cases. The apparatus used was most often the Esmarch-Skinner mask. A subcutaneous injection of morphia was generally given. In one of the fatal cases air entered a vein during removal of the thyroid body; in a case of removal of sequestrum from the femur, death occurred

[1] *Indian Medical Gazette*, Sept., 1891.

five and a half hours after the operation, partly from loss of blood and partly from the action of the chloroform on the heart; in a third, death occurred three hours after the operation, from obstruction of the respiratory passages by vomited material. In the other three fatal cases, fatty degeneration of the heart was found.

If ever collective investigation could be of any real service, it ought to be useful in such a case as this; that is, in ascertaining the percentage of deaths after the various anæsthetics. But even here it probably proves little; for we have no guarantee that all the members recorded their cases, and it is quite possible that a member having a larger percentage of deaths than usual might not care to publish his cases, while the member who had few deaths would be pleased to do so. The mortality given above is certainly small; and if it can be relied upon as approximately correct, it indicates a lower death-rate than we have attained to in this country.

W. H. Harsant.

The structure of the walls of sinuses is materially influenced by the cause and by the duration. Sir James Paget has thus described them: When they have existed for one or more years and are not inflamed, the walls are commonly hard, callous, not highly sensitive or easily bleeding, and formed of condensed connective tissue inseparable from the adjacent structures; in more recent cases, the walls are soft, like ordinary layers of recent granulations, sensitive, readily bleeding, and easily broken through. In diseased states they may be—as ulcers may be—inflamed, spongy, or œdematous, exquisitely sensitive or sloughing. The granulations lining the walls of sinuses vary with the differences in the walls themselves, being in recent cases coarsely granular and soft, and in old cases dense and firm, smooth on their free surface, with scarcely a trace of granular or papillary arrangement. The cells of the granulation tissue are either indistinguishable from pus cells or filled with fatty particles, and the pus is that of an unhealthy process until the sinus begins to heal.

Dr. C. S. Cole,[1] after giving the causes of sinuses—abscess, wound or gangrene, and ulceration—states that sinuses after amputation are either due to some fault in the minutiæ of the operation or to the patient's condition; insufficient drainage may cause a sinus, as may also a drainage tube retained for too long a time; but he has found it easier to close a sinus caused

[1] *American Practitioner and News*, August 1, 1891.

by a tube than one due to the lack of it. He states that if a sinus already exists, no form of injection is comparable to thorough curetting with a Volkmann's sharp spoon. If the wall is thick, either splitting it through and through, and scraping its sides, or actually dissecting it out in its entirety and making immediate approximation with sutures, or packing with gauze and using sutures in from 24—48 hours, are the best plans. Rest of the part is always an important adjunct to other measures, with general tonic treatment. If a sinus after amputation resists the above measures, the source of irritation must be removed (not always an easy matter), and a sufficient incision, even to the extent of removing the whole cicatrix, be made and the bone then carefully trimmed up. Then firm pressure should be made with gauze dressings, or a sponge wrung out of a hot carbolic solution; every effort should be made by careful trimmings not to leave to the tissues the disposition of any little fragments of bone. Thorough drainage with a full-sized tube should be provided for the first few days, and afterwards light gauze packing will answer.

A solution of chloride of zinc, 40 grains to an ounce, as recommended by Sir J. Lister, swabbed over the wound or round the sinus after scraping, will be found very useful. It is an antiseptic, the powers of which appear to last in the wound for two or three days, a slight hæmostatic and a slight stimulant to the formation of granulations.

W. J. PENNY.

### OBSTETRICS.

Many men in active practice have looked askance on the introduction of the antiseptic routine into their midwifery work, because the details recommended have appeared to them either unnecessary or impracticable. But the good results obtained of late years in hospital midwifery practice cannot be ignored; and it is only reasonable to conclude that some improvements might take place in private practice if methods could be devised for establishing asepsis which would not be unduly finikin, or which could be carried out by the average attendant, and with the appliances of the ordinary lying-in room.

A review of the recent literature of this subject leads to the hope that a *via media* may be found in which the general practitioner may reap the benefits of that asepsis which promises to dominate every department of medical work.

Fatal puerperal septicæmia, fortunately not of very frequent occurrence, is however so full of horrors to all concerned that anything which can obviate it will be cheerfully undertaken, even if it seems to involve unnecessary trouble; and as, short of death, there arise many septic troubles whose advent it is impossible to foresee, and which it is said are avoidable, the antiseptic practice must be carried out as a matter of routine.

Dr. Turner Anderson[1] says that puerperal septicæmia is due to contamination from external sources, and that auto-infection is probably a myth. He considers that sufficient evidence exists to prove that the *materies morbi* of puerperal sepsis may be carried to the lying-in woman from various sources, that the disease is in no sense a specific fever, and is certainly preventable. He says that before the days of antiseptic midwifery, at least five women out of every hundred confined in New York died of the puerperal state, but that now the mortality in hospital obstetric practice is almost *nil*. He attributes the good results to the altered mode of conducting labours, and cites, as an example, the practice of the Sloane Maternity Hospital of that city in a thousand consecutive cases. From an extensive personal experience, he considers that the measures and precautions there adopted may be applied in private practice, not only preventing puerperal fever, but appreciably favouring convalescence, which is often retarded by such troubles as subinvolution or pelvic cellulitis.

Dr. Archibald Dixon,[2] who deals more fully with the etiology of puerperal septicæmia, quotes other statistics which confirm those already given. After a narration of the routine adopted in Maternity hospital practice, which he admits is out of the question for the general practitioner, he lays down certain rules for private practice, by obedience to which he considers puerperal septicæmia can be stamped out, and child-bed fever become a thing of the past.

At Bonn, in May, at the meeting of the German Gynæcological Association,[3] Dohrn of Königsberg and Ahlfeld of Marburg read papers on the subject of "Private Obstetric Practice" and an interesting discussion followed, which showed that puerperal mortality and morbidity is much lower in institutions than in private practice. Dohrn uttered a strong word of warning against trusting too much to the antiseptic protection in cases of operative interference. He

[1] *American Practitioner and News*, June 20, 1891, p. 393.
[2] *Cincinnati Lancet-Clinic*, June 20, 1891, p. 755.
[3] *American Journal of Obstetrics*, August, 1891, p. 990.

said that since the introduction of antisepsis, operative deliveries had multiplied rapidly, but that neither the mother nor the child had been benefited. The enormous increase in operative deliveries in the Grand Duchy of Baden has not diminished the mortality of puerperæ there; and as for the kingdom of Saxony, he is even able to prove that among the deceased puerperæ from 1883 to 1889, the number of those delivered by operation has increased largely—that is to say, that operative deliveries there had to be charged from year to year with a larger proportion of puerperal deaths. In reference to the etiology of puerperal fever, Ahlfeld considers that as some deaths and many grave diseases still occur despite the most painstaking cleanliness, we are forced to assume auto-infection for some cases, but that in private practice the greatest number of deaths and severe diseases are due to direct infection. He presented statistics of 2,000 cases observed in the Marburg Maternity from April, 1884, to April, 1891.

Dr. Chauncey Palmer [1] records the diminution that has taken place in the mortality in the obstetric wards of the Cincinnati Hospital since the introduction of the antiseptic method, and gives the details of the practice adopted there.

From these communications to which I have referred, something like the following may be recommended as a routine mode in private practice:—The medical attendant must be constantly on the watch to see, not only for himself, but also for those who may touch the patient, that the most scrupulous cleanliness is observed. If it is possible the parturient woman should receive a vaginal injection of a quart of warm water, containing 1 in 2,000 of perchloride of mercury.[2] The hands and arms of the doctor should be thoroughly washed with soap and water, the nails receiving very special attention from a nail-brush. The hands are then to be immersed in a solution of perchloride (1 in 1,000), and an examination made with the fingers anointed with carbolised vaseline (1 in 40), or with lanoline or vaseline having a grain of perchloride to each ounce, or with glycerine containing 1 in 2,000 of the perchloride.[3] Vaginal examinations should be as few as possible. The writers insist upon this with much force; but in the discussion at Bonn, Frommel

[1] *International Clinics*, Volume Second, 1891, p. 164.

[2] Messrs. Ferris and Co., Bristol, issue a conveniently portable and cheap case of sublimate powders, prepared in accordance with the suggestions of Dr. Cullingworth.

[3] Herman, *First Lines in Midwifery*, 1891, p. 67.

and J. Veit pointed out that in clinical instruction it was not possible to restrict internal examinations, and that in their practice good results had been nevertheless obtained, although these no doubt may be partly attributed to the very careful asepsis observed. The third stage of labour should be left, if possible, entirely to nature, as it is bad practice to invade the vagina after delivery of the child, unless absolutely necessary. The old form of napkin placed against the vulva should be discarded, and there should be substituted for it a dusting with iodoform, or, perhaps better still, with aristol,[1] and a pad of gauze wrung out in hot perchloride solution of the strength of 1 in 4,000, and changed every six hours, or a large pad made of gauze filled with absorbent cotton, and not allowed to remain for the first day more than four hours at a time; or, failing these, the wood-wool diapers should be used. Everything about the bed which has become in the least soiled is to be removed. All wounds of the external genitals are to be closed by suture, and in all operative deliveries, whether of child or placenta, the most thorough attention is necessary to ensure asepsis. It is of the greatest importance to see that nurses and other attendants are very careful about all details, and that by their carelessness the doctor's work is not undone.

Ahlfeld considers that on these points the public should be instructed by oral and written communications; that the use of the thermometer should be obligatory in protracted labours; and that nurses and midwives should be compelled to record the temperature twice daily.

If lives of mothers are to be saved by rigid attention to these details, it is obviously necessary that the women in this country who attend most of the midwifery should be of a higher order of intelligence than those with whom we are now practically acquainted, and something must be done in the way of legislation to prevent a woman entering on the responsible duty of midwifery practice with no other qualification than that of poverty or incapacity for earning a livelihood in any other walk of life.

When the profession has become comfortably assured of the antiseptic virtues of the perchloride of mercury, there comes a rude shock in the shape of a communication from Dr. A. C. Abbott,[2] casting doubts upon its germicidal power. Experiments made in the pathological laboratory of the

[1] See *American Journal of Medical Sciences*, July, 1891, p. 81.
[2] See *American Journal of Medical Sciences*, July, 1891, p. 75.

Johns Hopkins Hospital led him to the following conclusions, somewhat similar to those of other observers:—

> That, under the most favourable conditions, a given amount of sublimate has the property of rendering inert only a certain number of individual organisms.
>
> That the disinfecting activity of the sublimate against organisms is profoundly influenced by the proportion of albuminous material contained in the medium in which the bacteria are present.

He, therefore, considers that there are two serious objections to the employment of sublimate solutions upon wound-surfaces: (1) the albumin of the tissues and fluids of the body tends to diminish the strength of, or indeed renders entirely inert, the solution employed; (2) the integrity of the tissues, which in their normal condition possess the power of rendering inert many kinds of organisms which gain access to them, is materially injured by the application of this salt, which brings about a condition of superficial necrosis. But as Dr. Abbott admits that as a disinfectant in the strict sense of the word, there are perhaps few substances which possess the property in a higher degree than does corrosive sublimate, it will be wise for us, till further negative evidence is forthcoming, to rely upon the methods which have been attended with such beneficent results.

*　*　*　*

In connection with this subject of puerperal septicæmia, careful attention should be given to a paper, by Dr. Robert Boxall,[1] on "Fœtor of the Lochial Discharges," in which is presented an analysis of 640 cases admitted to the General Lying-in Hospital. Dr. Boxall draws particular attention to the following points:—

1. That septic infection may take place without fœtor.
2. That fœtor may occur without sepsis or fever.
3. That fœtor is more frequent in cases where the tissues are bruised and torn, and therefore in primiparæ and in operation cases.
4. That fœtor is generally (though not invariably) associated with fever, but in such cases the fever almost always precedes the fœtor by a considerable interval.
5. That the presence or absence of fœtor is a very uncertain guide to the presence or absence of sepsis.

[1] *Practitioner*, May, 1891, p. 330.

6. That in any case, as fœtor invariably indicates a failure to maintain local asepsis, vigorous antiseptic measures should be at once instituted.
7. That the vulva and vagina should be first cleansed, and only when this has been done, and where real necessity exists, should the cleansing be extended to the interior of the uterus.

L. M. Griffiths.

### LARYNGOLOGY.

At the beginning of the year 1891, laryngologists were hopeful that Professor Koch had discovered a specific for that terrible disease laryngeal tuberculosis. It is with much disappointment that we are obliged to confess that tuberculin (Kochii) has been tried in the balance and found wanting. The indictment made against its use by Virchow, in the long and noted discussion by the Berlin Medical Society,[1] on the ground that it had stirred up fresh tubercular infiltration in the bodies of people who were suffering from chronic and more or less quiescent tuberculosis, has been supported by the observations of many others. It has also been clearly shown that we are unable to determine, even now, what case is liable to this dangerous awakening of fresh mischief. All who have worked with the drug must be conscious of this serious drawback to its use, and I have seen striking examples of it. Even now, however, some leading German specialists administer it, and, as they state, with benefit to their patients, and "cured cases" are still published. Grabower[2] has treated 38 cases of laryngo-pulmonary phthisis, with the result that 5 were cured, 22 improved, 6 remained unaffected, 5 became worse. Michelson[3] reports 3 cases of lupus of the throat and nose, of which 2 were cured and 1 improved. Professor B. Fränkel, who was such a doughty champion of tuberculin in the early months of the year, has, so far as any public statement goes, not renounced his allegiance; and others in Germany, whom we must regard as authorities, are still keeping their minds open as to its value. Cantharidin (Liebreich) has not gained many friends, although, like tuberculin, it was looked upon very favourably at first, as a drug that would cure tuberculosis.

[1] *Berl. klin. Woch.*, Dec. 1890, Jan., Feb., and March, 1891.
[2] *Deutsch. med. Woch.*, Nos. 23 to 28, 1891.
[3] *Journal of Laryngology*, July, 1891.

Diphtheria always occupies much space in laryngological literature; and evidently treatment by mercurials, by insufflation, gargles, sprays, inunction and internally, holds the field. One novelty has been introduced by Seibert,[1] who injects, with a suitable hypodermic syringe, 12 to 15 drops of chlorine water into and through the false membrane, in the belief that the pathogenic bacillus exists in that situation, and that it can be destroyed in this way. Guthrie,[2] Rozenzweig,[3] and Beale [4] all report favourably of the treatment of post-diphtheritic paralysis by injections of strychnine.

There have been a great many examples of the widespread influence of the nasal reflexes published. All who are in the habit of examining the nose carefully are, of course, aware of the importance of not forgetting these reflexes, and of the frequency with which we meet with pain in the head, etc., eye troubles, asthma, etc., which are due to nasal abnormality. I rarely find an asthmatic patient who has a reasonably normal nose,—*i.e.*, a septum, straight and free from spurs, and turbinated bodies that are of healthy size and reflex mobility; and I have cured conditions dependent on this nasal trouble by proper treatment of the nose. But, as was abundantly proved by a recent discussion on asthma by the Medical Society of London, we cannot say what case of asthma will be benefited, and what case remain unaffected, by treating the nasal mischief. We must therefore be careful not to prophesy cure of asthma after removal of nasal mischief; although we ought always to do what is necessary in this direction.

Moure and Bergoiné [5] strongly advocate electrolysis for treating deviations and spurs of the nasal septum, as being very successful and not nearly so unpleasant to the patient as the other methods of treatment by saw, chisel, or trephine. This plan of removing these growths and deformities has the great advantage of giving rise to no open wound in the nose, and of sparing the mucous membrane; although I have never seen any trouble result from a clean cut with Bosworth's saw or from the use of the galvano-cautery in these cases.

Aristol is used with success in the treatment of atrophic rhinitis and syphilitic ulcer of the nose; and spite of Heller's statement [6] that it is less powerful in its action on bacteria

[1] *New York Med. Journ.*, Dec. 6, 1890. [2] *Lancet*, April 18, 1891.
[3] *Lancet*, May 9, 1891. [4] *Brit. Med. Journ.*, May 30, 1891.
[5] *Journal of Laryngology*, Dec., 1890.
[6] *Berl. klin. Woch.*, May 4, 1891.

than iodoform or even iodol, I have found it of service in such cases.

A word in season as to the dangers of the nasal douche has been uttered by Loewenberg.[1] If it be faultily used, either as regards the position of the head or the force employed, or if we prescribe it in cases with adenoid growths in the post-nasal space, or stenosis of the nostrils from deviations of the septum, polypi, or hypertrophied turbinated bodies, we may get serious results as regards the middle ear. A nasal douche ought to be prescribed only after a careful examination of the nasal and post-nasal passages; and then it should not be a syphon douche, but one under the most complete control of the patient, and should throw five small streams and not one large one. In my experience, nasal douches are much too frequently employed by patients, and very inefficient instruments are pressed into service.

B. J. Baron.

### Reviews of Books.

[1] *Berl. klin. Woch.*, May 4, 1891.

